# Regeneration of Buccal Wall Defects after Tooth Extraction with Biphasic Calcium Phosphate in Injectable Form vs. Bovine Xenograft: A Randomized Controlled Clinical Trial

**DOI:** 10.3390/dj11090223

**Published:** 2023-09-19

**Authors:** Marija Čandrlić, Matej Tomas, Marko Matijević, Željka Perić Kačarević, Marijana Bićanić, Žarko Udiljak, Ivana Butorac Prpić, Ivan Miškulin, Slavko Čandrlić, Aleksandar Včev

**Affiliations:** 1 Department of Dental Medicine, Faculty of Dental Medicine and Health Osijek, Josip Juraj Strossmayer University of Osijek, 31 000 Osijek, Croatia; marija.candrlic@fdmz.hr (M.Č.); mtomas@fdmz.hr (M.T.); marijana.bicanic@poliklinikaminerva.hr (M.B.); zarko.udiljak@gmail.com (Ž.U.); butoracivana88@gmail.com (I.B.P.); 2 Community Healthcare Center of Osijek-Baranja County, 31 000 Osijek, Croatia; oralnakirurgija.osijek@gmail.com; 3 Department of Anatomy, Histology, Embriology, Pathology Anatomy and Pathology Histology, Faculty of Dental Medicine and Health Osijek, Josip Juraj Strossmayer University of Osijek, 31 000 Osijek, Croatia; zpkacarevic@fdmz.hr; 4 Botiss Biomaterials GmbH, 15806 Zossen, Germany; 5 Department of Public Health, Faculty of Medicine Osijek, Josip Juraj Strossmayer University of Osijek, 31000 Osijek, Croatia; imiskulin@mefos.hr; 6 Department of Interdisciplinary Areas, Faculty of Dental Medicine and Health Osijek, Josip Juraj Strossmayer University of Osijek, Crkvena 21, 31 000 Osijek, Croatia; scandrlic@fdmz.hr; 7 Department of Pathophysiology, Physiology and Immunology, Faculty of Dental Medicine and Health Osijek, Josip Juraj Strossmayer University of Osijek, 31 000 Osijek, Croatia

**Keywords:** histology, biphasic calcium phosphate, bovine xenograft, fenestration, guided bone regeneration

## Abstract

Bone healing after tooth extraction may be affected by defects of the alveolus buccal wall, such as fenestrations and dehiscences. Therefore, to minimize dimensional changes it is advisable to perform alveolar ridge preservation after tooth extractions. Different biomaterials are used for this purpose. The aim of this study was to investigate the qualitative and quantitative histological changes in human biopsies taken after 6 months of healing of extraction sockets with buccal wall defects. For this purpose, the defects of 36 patients (18 per group) were treated with injectable biphasic calcium phosphate (I-BCP) or bovine xenograft (BX) after extraction. After six months of healing, biopsies were taken and proceeded to the histology laboratory. No evidence of an inflammatory response of the tissue was observed in the biopsies of either group, and the newly formed bone (NB) was in close contact with the remaining biomaterial (BM). The histomorphometric results showed that there was no statistically significant difference between the groups in the mean percentage of NB (*p* = 0.854), BM (*p* = 0.129), and soft tissue (*p* = 0.094). To conclude, both biomaterials exhibited osteoconductivity and biocompatibility and achieved satisfactory bone regeneration of buccal wall defects after tooth extraction.

## 1. Introduction

Dental implants are used as an alternative to partial or complete dentures and fixed dental prostheses. It can be said that dental implants are the gold standard when it comes to replacing one or more lost teeth, which is confirmed by clinical studies that have shown that this is the most convenient therapeutic solution for patients to solve the problem of partial or complete edentulism [1,2]. Moreover, studies have shown that dental implants are the optimal solution for the treatment of edentulism from the aesthetic and functional points of view [3].

However, following tooth extraction bone undergoes dynamic changes of the resorption and de novo bone formation, which is under physiological conditions a balanced process, but after tooth extraction, the resorptive processes in bone predominate over the processes of new bone formation, resulting in a reduction of the vertical and horizontal dimensions of the alveolar ridge [4,5]. Remodeling of the alveolar ridge is a progressive process. In the first year after extraction an average of 25% of the alveolar bone volume is lost and after three years this increases up to 40%. Complicating factors in the process of bone remodeling that can lead to even greater dimensional changes in the alveolar ridge during healing is the presence of bone dehiscence and fenestrations [6].

Dehiscence and fenestration are defects of the vestibular bone wall that are usually the result of a thin buccal wall, periapical or periodontal pathology, or trauma during extraction [7]. As already mentioned, dehiscence and fenestration can be disruptive factors in bone healing after tooth extraction, since the volume loss is greater in the molar region and on the vestibular walls of the alveolus [5,8]. Therefore, in order to minimize dimensional changes of the alveolar ridge it is advisable to perform a surgical procedure such as ridge preservation [9].

However, the success of augmentation procedures as well as overall implant-prosthetic rehabilitation depends not only on the choice of a surgical technique and the clinical skills of the provider, but also on the knowledge and proper selection and use of the various biomaterials. The science of the dental biomaterials is one of the fastest growing disciplines in dentistry, and a more detailed knowledge of the properties of existing biomaterials and the development of new ones are certainly areas that require special research attention. Biomaterials for bone regeneration are divided into bone substitutes (allografts, xenografts, and alloplastic bone materials) and membranes [10].

Xenografts are bone substitutes derived from a genetically completely different species than the host. They are usually obtained from animals, primarily cattle, pigs, and horses. To remove the organic component, the graft is mechanically and chemically processed before use. Removal of the organic component is necessary to prevent a host immune response to the foreign body. In addition, this process reduces the possibility of transmission of infectious diseases [11]. The safety, biocompatibility, and osteoconductivity of bovine xenograft (BX) have been described in clinical, histologic, and radiologic studies in humans [12,13,14,15,16,17,18].

Alloplastic bone substitutes or alloplasts are completely synthetic biomaterials, so their availability does not depend on a living individual. The group of calcium phosphate biomaterials is particularly interesting because their chemical composition is very similar to human bone, they have excellent osteoconductive properties, and they form a very strong bond with bone during bone remodeling [19]. The term biphasic calcium phosphate (BCP) was first used by Ellinger et al. [20] to describe a ceramic material consisting of a combination of hydroxyapatite (HA) and beta-tricalcium phosphate (β-TCP). The initial description of the mixture was based on X-ray diffraction analysis, which revealed 20% of HA and 80% of β-TCP in the analyzed biomaterial. This combination showed significant advantages compared to other calcium phosphate-based biomaterials. Today, HA and β-TCP are mixed in different ratios, usually 60:40 and 70:30, and it is the different ratios of these compounds that determine the bioactivity and biodegradability of the material [20,21,22].

Injectable biphasic calcium phosphate (I-BCP) showed good results in augmentation of three-dimensional cavities such as the maxillary sinus, extraction wounds or intrabony periodontal defects [22,23,24,25,26]. It is characterized by its viscosity, which allows more complete and easier filling of the bone defects. In addition, the material does not need to be mixed with physiological solution or blood. Overall, the use of I-BCP shortens the duration of the surgical procedure and therefore reduces the discomfort for the patient [27]. As mentioned earlier, vestibular wall defects could be an aggravating factor for bone healing after tooth extraction. Therefore, we aimed to compare regenerative potential of I-BCP, based on histologic evaluation 6 months after treatment of vestibular wall defects, with the well-known bovine xenograft (BX).

## 2. Materials and Methods

### 2.1. Ethics Statement

The study was approved by the Ethics Committee of the Osijek-Baranja County Health Centre (Reg. No.: 03-1897/20) and the Faculty of Medicine of the Josip Juraj Strossmayer University of Osijek (Class: 602-04/22-08/02, Reg. No.: 2158-61-46-22-109). The randomized controlled trials registration number was also provided by ClinicalTrials.gov (NCT06020092). The identity of patients participating in the study was fully protected. Prior to enrollment in the study, all study participants were provided with detailed written and verbal information about the objectives and methodology of the study and the methods of dissemination of study results. All study participants voluntarily signed an informed consent form as one of the conditions for participation in the study.

### 2.2. Study Design, Inclusion and Exclusion Criteria

The study was designed as a randomized controlled clinical trial. To participate in the study, patients were required to have at least one tooth predisposed for extraction and to have a dental implant placed after completion of the healing period. The indications for tooth extraction were: chronic periapical process, chronic periodontal disease, or root fracture. The inclusion criteria were: age between 18 and 60 years, intact physical and mental health, understand of the study protocol and signed an informed consent. Participants were recruited in July and August 2021.

Patients were excluded from the study if they had at least one of the absolute contraindications to implant prosthetic therapy described by Wang and Hwang [28]. Other exclusion criteria included the following systemic diseases: uncontrolled diabetes, osteoporosis and osteopenia, and vitamin D deficiency, bisphosphonate therapy, glucocorticoid therapy, hypothyroidism, uncontrolled cardiovascular disease (hypertension, coronary artery disease, congestive heart failure), and the following local factors: use of tobacco products (up to 10 cigarettes per day) and poor oral hygiene. Pregnant and lactating women were not included in the study [29].

In addition to the general health criteria mentioned above, the patient had to meet at least one intraoral criterion, namely the presence of a buccal bone defect such as fenestration or dehiscence. Patients who met the inclusion criteria and were willing to participate, were randomly assigned via a web interface (https://www.randomizer.org/ accessed on 28 April 2021) into test and control group [30].

### 2.3. Participants and Materials

Participants were recruited from the oral surgery office. A total of 36 healthy patients were included in the study. In analyzing the validity of the test, the approach of equivalence of the tested methods and noninferiority of the tested method compared with the existing method was used. With a significance level α of 0.05, a test power of 80%, an expected standard deviation of the predicted result of 10, and an equivalence limit of d = 10, it was necessary to include at least 36 subjects in the study, 18 subjects per group [31,32].

A total of 18 patients in the test group received I-BCP (maxresorb^®^ inject, botiss GmbH, Berlin, Germany). This biomaterial is an alloplastic bone substitute material in the form of a paste that is placed into the defect with a plastic syringe. The material consists of an aqueous gel containing granules of biphasic calcium phosphate in the composition of 60% HA and 40% β-TCP (particle size between 15–50 nm). Patients in the control group received BX (cerabone^®^, botiss GmbH, Berlin, Germany). The material is completely anorganic and consists of 100% HA. For this study, the material was used in granular form (the size of the granules is between 0.5 and 1 mm), mixed with physiological solution prior to placement into the defect. The randomisation process was carried out by one person (M.Č.). Blinding was not possible because I-BCP was administered in the form of a paste, while BX in the form of a granulate was mixed with a physiological solution before administration.

### 2.4. First Surgical Phase and Healing Period

**Figure 1:**
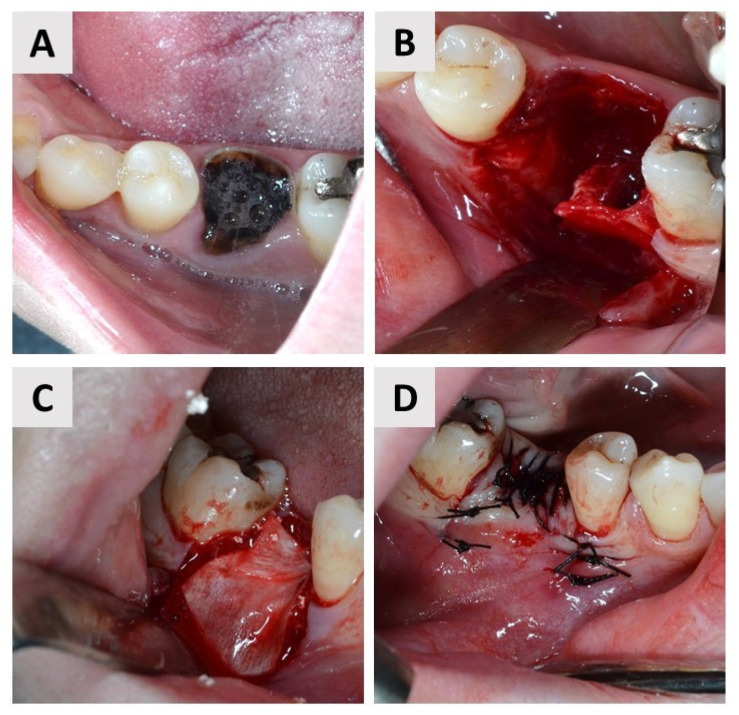
First surgical phase—test group. (**A**) A tooth 46 (FDI notation system) was selected for extraction. (**B**) The tooth was extracted atraumatically. Note the defect in the buccal bone wall. (**C**) The alveolus was filled to the margin with I-BCP and covered with resorbable membrane. (**D**) Primary wound closure with single sutures.

**Figure 2:**
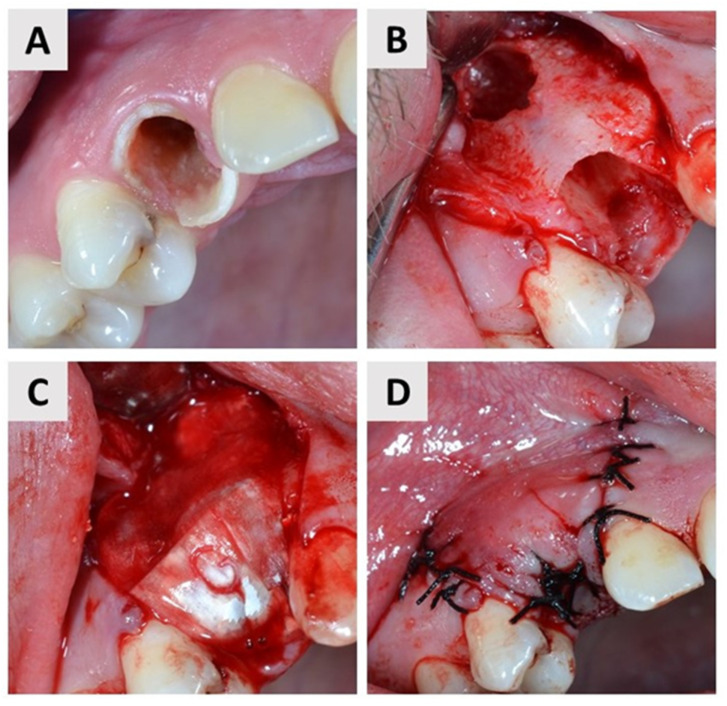
First surgical phase—control group. (**A**) A tooth 13 (FDI notation system) with the extensive caries. (**B**) Alveolus after atraumatic extraction and curretage. Note the fenestration in the buccal bone wall. (**C**) The alveolus was filled to the margin with BX and covered with a resorbable membrane. (**D**) The flap was adapted, and primary wound closure was achived.

Both surgical phases were performed by the same experienced oral surgeon (M.M.). Before the procedure, the patient was administered a 3% chlorhexidine solution to rinse the oral cavity for one minute. Local anesthesia (2% Lidocaine^®^, Belupo, Koprivnica, Croatia) was then administered and the surgical field was covered with sterile drapes. A full-thickness mucoperiosteal flap was elevated at the extraction site (Figure 1B and Figure 2B). To perform the tooth extraction atraumatically, the roots of the multirooted teeth were separated with a drill before extraction. The extraction was performed with atraumatic instruments, with as little trauma to the alveolus as possible. The alveolus was then carefully examined and curetted to remove granulations and stimulate fresh bleeding in the alveoli (Figure 1B and Figure 2B). The alveolus was then filled to the edge with I-BCP in the test group and BX in the control group. In the end, bone grafting biomaterial was covered with a resorbable native collagen membrane obtained from porcine pericardium (Jason^®^ membrane, Botiss GmbH, Berlin, Germany) in both groups (Figure 1C and Figure 2C). The mucoperiosteal flap was then adjusted and primary closure was achieved using single sutures (5.0 monofilament) (Figure 1D and Figure 2D). All phases of the first surgical procedure, separately by the groups, are shown in the sets of photographs taken during the surgical procedures (Figure 1 and Figure 2). The patient was then prescribed an analgesic (400 mg ibuprofen) and instructed to take antibiotics (amoxicillin with clavulanic acid or clindamycin) for the next 7 days. Patients were given standardized postoperative instructions for oral hygiene. They were instructed to brush their teeth with a soft brush and to make sure that the sutures remained free of food debris. They were also given a mouthwash with chlorhexidine gluconate 0.12% for 10 days and instructed to use it from the second postoperative day. On the 10th day after surgery, the sutures were removed.

A 6-month period of regeneration of the bone defect followed. During this period, the patient reported for follow-up examinations at the first, second, and third months after surgery and immediately before the sixth month to arrange for a second surgical procedure. Based on the radiographic examination, the selection and positioning of the appropriate implant was performed.

### 2.5. Second Surgical Phase

Six months after extraction, patients came to the office for placement of a dental implant (Ankylos^®^, Denstply Sirona, Charlotte, NC, USA). The preparation of the patient (rinsing of the oral cavity with an antiseptic solution, local anesthesia, sterile covering of the working area) is the same as for the first surgical procedure. Subsequently, a biopsy at pre-existing defect was taken using a trephine drill (Ustomed^®^ intrumente, Tuttlingen, Germany). The inner diameter of the trephine drill was smaller than that of the final drill used to prepare the implant bed, to avoid unnecessary removal of the patient’s bone. In addition, each biopsy was taken from the central part of the pre-existing defect to reduce the possibility of an incorrect biopsy (e.g., biopsy of only the native bone that was not treated with biomaterial).

### 2.6. Histological Analysis

The trephine drill was placed in a hermetically sealed plastic tube containing a 4% formaldehyde solution and sent to the histology laboratory for further analysis. The histological processing was the same as previously described by our group of authors [26]. The samples were stained using Masson’s trichrome and Movat’s Pentachrome.

Pathohistological analysis evaluated the tissue response to the implanted biomaterial, i.e., the presence of fibroblasts, blood vessels, neutrophils, monocytes/marophages, and multinucleated giant cells (MGC).

Histomorphometric processing of the specimens was performed using the free ImageJ software (https://imagej.nih.gov/ij/download.html accessed on 22 June 2022). The surface of the new bone, the surface of the biomaterial, and the surface of the soft tissue were marked on each specimen. The surface was then measured, and the following was calculated from the data obtained: percentage of new bone formed, percentage of biomaterial remaining, and percentage of soft tissue [24].

### 2.7. Statistical Analyses

Categorical variables are reported with absolute and relative frequencies. Differences between categorical variables were tested with the Fisher-Freeman-Halton test.

Continuous data are described by the arithmetic mean and standard deviation. The normality of the distribution was tested using the Shapiro-Wilk test. Differences in continuous variables were tested with the *t*-test (with corresponding difference and 95% confidence interval of the difference). All P values were two-sided. The significance level was set at alpha = 0.05. IBM SPSS Statistical software, version 27.0.1 (IBM Corp., Armonk, NY, USA), was used for the analysis.

## 3. Results

**Figure 3:**
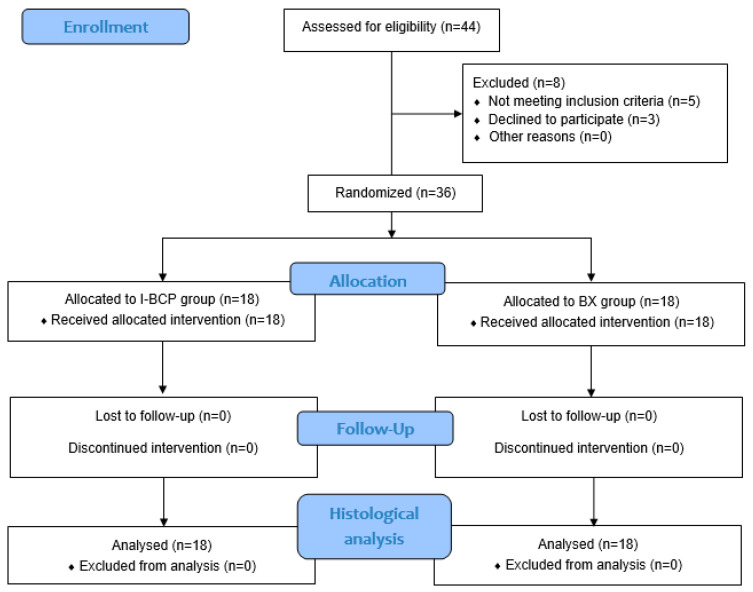
Study CONSORT Diagram.

A total of 44 oral surgery patients were included in the screening process for participation in the study. Of these, 36 patients fully met the inclusion criteria and signed an informed consent form to participate in the study (Figure 3). The reason for extraction was deep caries lesions with chronic periapical periodontitis in 32 patients and fractures of the tooth crown and/or root in the other 4 patients.

Each patient had only one extraction site, after which the wound was filled with biomaterial. There were no significant differences in the distribution of subjects according to gender (*p* = 0.505, Fisher-Freeman-Halton exact test) and age (*p* > 0.99, Fisher-Freeman-Halton exact test) (Table 1.).

The healing time was eventful in the patients of both groups. Only 4 patients in the test group and 3 patients in the control group reported mild side effects such as edema and pain at the surgical site. Edema and pain were treated locally with cold packs and systemically with previously administered oral antibiotics and analgesics. At the time of suture removal, edema and pain had resolved in all patients. No membrane exposure was observed in the postoperative controls. By the third month after ridge preservation, mucosa was healed at the extraction sites in all participants.

### 3.1. Quantitative Histological Findings

Quantitative histologic analysis was performed on a total of 36 biopsies (18 per group). No statistically significant difference was found between groups in the percentage of newly formed bone, residual biomaterial, and soft tissue (two-tailed *t*-test). A more detailed presentation of the histomorphometric results can be found in the Table 2.

### 3.2. Qualitative Histological Analysis

**Figure 4:**
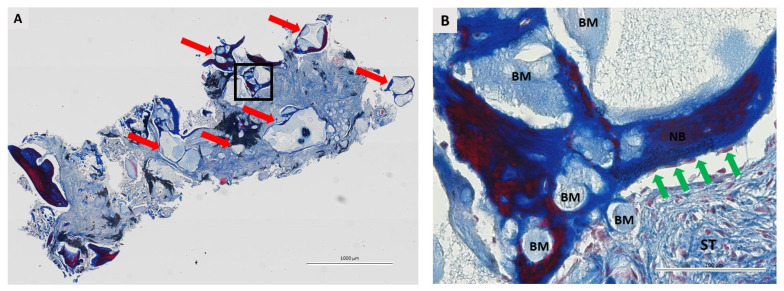
Bone biopsy six months after ridge preservation with I-BCP stained with Masson’s trichrome. (**A**) Longitudinal section. Along the entire specimen, residual biomaterial and newly formed bone (red arrows) can be seen in the area where bone regeneration occurs. The square indicates the area shown at higher magnification. Scale bar 1000 μm. (**B**) Granules of residual biomaterial (BM) are incorporated into the newly formed bone (NB) and surrounded by soft tissue (ST). Active osteoblasts (green arrows) indicate active bone remodeling. No signs of inflammatory reaction were noted. Scale bar 100 μm.

**Figure 5:**
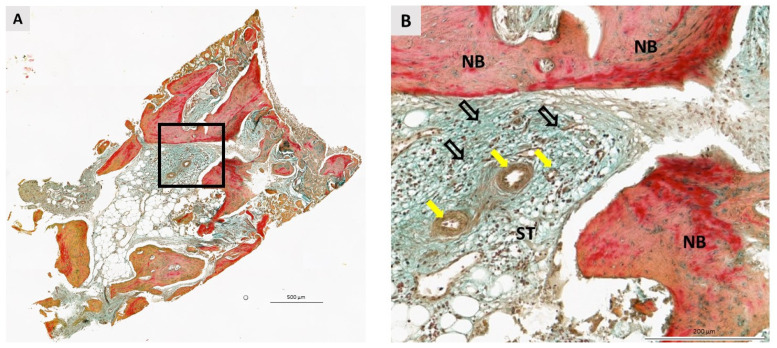
Bone biopsy six months after ridge preservation with I-BCP, stained with Movat’s Pentachrome. (**A**) Longitudinal section. The square indicates the area shown at higher magnification. Scale bar 500 μm. (**B**) The newly formed bone (NB) is surrounded by soft tissue (ST). Small blood vessels are easily detected (yellow arrows). ST is rich in fibroblasts whose nuclei are indicated by nofilled black arrows. No signs of inflammatory reaction of the tissue were found. Scale bar 200 μm.

**Figure 6:**
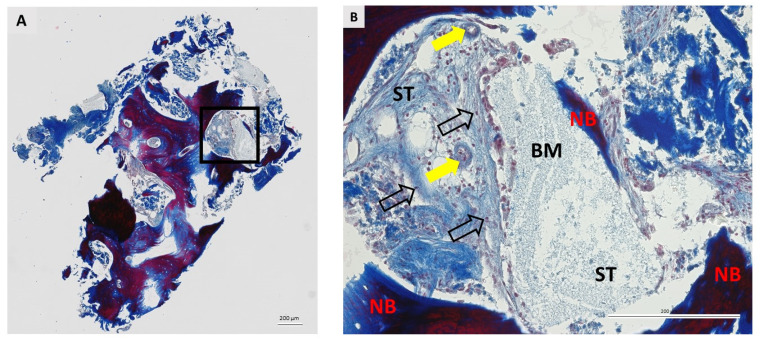
Bone biopsy taken six months after ridge preservation with BX and stained with Masson’s trichrome. (**A**) Longitudinal section. The square shows the area under higher magnification. Scale bar 100 μm. (**B**) Granules of residual biomaterial (BM) are incorporated into the newly formed bone (NB). Blood vessels (yellow arrows) and fibroblasts (nofilled black arrows) are in soft tissue (ST). No signs of inflammatory reaction were observed in the specimen. Scale bar 200 μm.

**Figure 7:**
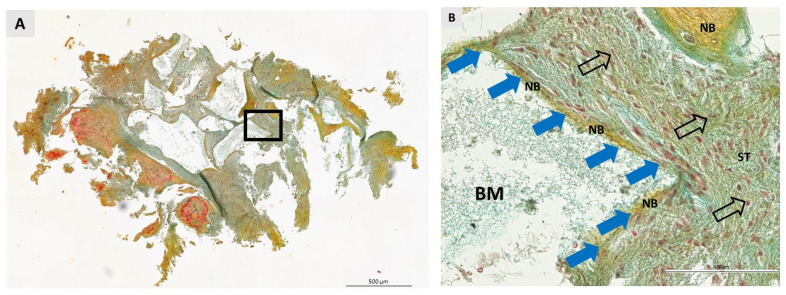
Bone biopsy taken six months after ridge preservation with BXand stained with Movat pentachrome stain. (**A**) Longitudinal section. The square shows the area under higher magnification. Scale bar 500 μm. (**B**) The image shows an apposition line (blue arrows) in direct contact with the remaining biomaterial (BM). The soft tissue (ST) is rich in fibroblasts, whose nuclei are indicated by nonfilled black arrows. No signs of inflammatory reaction were detected in the specimens. Scale bar 100 μm.

A total of 36 biopsies, 18 per group, were included in the pathohistological analysis. The same pathohistological changes were observed in both groups (Figure 4, Figure 5, Figure 6 and Figure 7). Newly formed bone (NB) was in close contact with residual biomaterial (BM). Vascularized, nonmineralized soft tissue (ST) is clearly visible in the bone biopsies. Successful bone regeneration is evident throughout the specimen. Bone regeneration occurs specifically at the point of contact between BM and the surrounding tissue, in the apposition line. The apposition line is a thin line along which osteoblasts are located, active bone cells that indicate bone remodeling. NB has a regular structure with osteocytes located in lacunae, which is a characteristic of lamellar bone. BM has an irregular appearance and is incorporated into NB and non-mineralized tissue. Fibroblasts are the most numerous cells of ST. No multinucleated giant cells were observed in the specimens after 6 months of healing. Also, cells characteristic of an inflammatory response in the tissue were not observed in any sample, indicating the biocompatibility of both the biomaterials and the surrounding tissue.

## 4. Discussion

Tooth extraction results in qualitative and quantitative changes in the soft and hard tissues of the alveolar ridge. In addition, buccal wall defects such as dehiscence and fenestration, which are usually the result of chronic periapical and/or periodontal lesions or trauma during extraction, may further impair alveolar healing. To reduce these changes, it is recommended after tooth extraction to preserve the alveolus according to the principles of guided bone regeneration (GBR), a surgical technique that has been shown to prevent alveolar bone volume loss after extraction [33,34,35]. This study was conducted as a randomized controlled clinical trial evaluating the qualitative and quantitative histologic changes in bone biopsies taken six months after ridge preservation of the postextraction alveolus with buccal fenestration and dehiscence with two different biomaterials.

The mean age of patients in the test group was 36.0 ± 10.4 years and in the control group 36.9 ± 13.4 years, with no statistically significant difference in the age of patients between groups (*p* = 0.505, Fisher-Freeman-Halton exact test). Previous studies have shown that patient age affects bone regeneration potential. Bone healing is indeed a complex biological process in which inflammation occurs initially, with various cells interacting in the microenvironment, mainly the monocyte-macrophage lineage, then osteoclasts, mesenchymal stem cells, and osteoblasts. With age, inflammatory processes increase, which may impair osteogenesis [36,37,38]. For this very reason, only patients aged 18 to 60 years were included in the study to minimize the above-mentioned factors affecting osteogenetic processes. No significant difference was found between patients in the test and control groups with respect to gender (I-BCP: 39% female and 61% male vs. BX: 55% male and 45% female; *p* > 0.99, Fisher-Freeman-Halton exact test). Overall, the distribution of participants by gender and age is consistent with previously published similar studies [24,25,39,40].

Histologic analysis of bone biopsies is neither common nor routine in clinical work with dental biomaterials. However, monitoring bone healing by histological analysis is extremely valuable as it reveals differences in the quantity and quality of newly formed bone depending on the type of biomaterial used for augmentation [41]. Pathohistological analysis of the bone biopsies showed that the biomaterial in both groups was fully integrated into the surrounding tissue and was in close contact with the newly formed bone. These observations are identical to those previously described in human histological studies in which various bone defects were augmented with BX [42,43] or I-BCP [23,24,25]. Close contact between the residual biomaterial and the newly formed bone was observed in the biopsies of both groups, suggesting the osteoconductive properties of the biomaterial [44]. After 6 months of healing, no MGCs, cells indicative of an immune response to a foreign body, were found in the biopsies of either the test or control group [45]. The role of these cells in bone regeneration and biomaterial degradation has not been fully elucidated [46,47]. However, it has been shown that higher numbers of MGCs were found in biopsies where healing was compromised, especially with respect to early exposure of the biomaterial to the oral cavity [48]. Clinical observations during the healing phase showed that there was no exposure of the biomaterials in the oral cavity in the test and control groups. In addition, patients reported only mild side effects in the form of edema and pain at the surgical site, and no complications that could affect healing were observed during follow-up. Also, cells characteristic of an inflammatory reaction of the tissue were not observed in any of the samples examined, indicating the biocompatibility of both biomaterials with the surrounding tissue [49,50].

Histomorphometric analysis showed a mean 31.5 ± 13.24% of newly formed bone in the test group. It can be said that the reported result is in accordance with the results of similar histological studies with BCP or I-BCP, in which about one third of the specimen was newly formed bone, such as in the studies of Jelušić et al. [51], Mangano et al. [52], Čandrlić et al. [25], Tomas et al. [24] and Schmitt et al. [42]. The same findings were observed with the mean percentage of the residual biomaterial and soft tissue in the test group.

A literature search revealed a number of comparative histological studies on the regenerative potential of I-BCP and BX, mainly on the maxillary sinus model. In the study by Oh et al. [53] histomorphometric analysis 6 months after maxillary sinus elevation with BCP and BX, showed that there was no significant difference in histomorphometric results between the groups. In the study by Wagner et al. [54] focus was on the comparison between BCP and BX mixed with autogenous bone. The results of their study also showed that both biomaterials had comparable histomorphometric and clinical outcomes. A randomized clinical trial comparing I-BCP and BX was also published by Kraus et al. [55]. In this study, both biomaterials showed comparable results in histomorphometric analysis of newly formed bone between groups. However, a significantly higher percentage of soft tissue was observed in the biopsies of the BCP group compared to the BX group. Cordaro et al. [56] also came to the same conclusions in their study. Previous studies do not indicate whether the percentage of soft tissue in the biopsies is clinically relevant, so it would be interesting to investigate in further studies whether this difference has clinical implications. Although this remains unknown for now, it is known from previous studies that the quality and quantity of regenerated bone and the absence of an inflammatory reaction of the tissue are important prerequisites for the long-term success of implant prosthetic therapy [57,58]. Our group of authors previously reported two histologic studies on the osteogenic potential of I-BCP compared with BX in different clinical indications. Interestingly, the average percentage of soft tissue was significantly higher when I-BCP was used to preserve the intact alveolus after extraction [25]. In contrast, histologic analysis showed no statistically significant differences between groups when the same biomaterial was used to treat alveoli with at least one wall defect after extraction [24]. In the present study, we obtained the same results. This confirms that the type of defect is an important factor in the regenerative potential of the biomaterial, so this study certainly represents an important update of the current knowledge on the osteogenic potential of I-BCP in different clinical indications.

It could be argued that the lack of a negative control group, in which biopsies were taken after 6 months of healing without intervention, is a limitation of the present study. However, several studies have confirmed that alveolar preservation minimizes bone resorption compared with healing without intervention [59,60]. Therefore, no negative control was included in the study. Harvesting bone from the regenerated site can be challenging as it is not a standard procedure in oral surgery. In addition, bone harvesting is performed simultaneously with the placement of the dental implant, so care must be taken to ensure a favourable implant position during bone harvesting, which may lead to incorrect harvesting.

In conclusion, for both biomaterials studied it was confirmed osteoconductive properties, biocompatibility and safety for treatment of buccal bone wall defects. The percentage of the newly formed bone is satisfactory in both groups. Still, there is a room for new studies on the immunological response to I-BCP as well as on its use in various clinical indications.

## Figures and Tables

**Table 1:** Demographic data of participants.

	I-BCP ^1^	BX ^2^	*p*-Value
Gender			
Female	7 (39%)	10 (55%)	0.505
Male	11 (61%)	8 (45%)	
*n*	18	18	
Age (years)			
Mean	36.0	36.9	
SD	10.4	13.4	>0.99
Min	20	18	
Max	55	59	

^1^ Injectable biphasic calcium phosphate, ^2^ Bovine xenograft.

**Table 2:** Histomorphometrical results.

	Newly Formed Bone (NB)	Residual Biomaterial (BM)	Soft Tissue (ST)
IBCP ^1^	31.5 ± 13.24%	14.01 ± 10.81%	54.48 ± 9.57%
BX ^2^	32.42 ± 16.02%	18.88 ± 11.66%	48.69 ± 12.09%
*p*-value *	0.854	0.129	0.094

^1^ Injectable biphasic calcium phosphate, ^2^ Bovine xenograft; * two-tailed *t*-test.

## Data Availability

The data presented in this article are available on request from the corresponding author.
